# The ASEAN economic community and medical qualification

**DOI:** 10.3402/gha.v7.24535

**Published:** 2014-09-10

**Authors:** Jathurong Kittrakulrat, Witthawin Jongjatuporn, Ravipol Jurjai, Nicha Jarupanich, Krit Pongpirul

**Affiliations:** 1 Medical Students for Health Systems and Services (MS-HSS), Thailand Research Center for Health Services System (TRC-HS), Faculty of Medicine, Chulalongkorn University, Bangkok, Thailand; 2 Department of Preventive and Social Medicine, Faculty of Medicine, Chulalongkorn University, Bangkok, Thailand; 3 Department of International Health, Johns Hopkins Bloomberg School of Public Health, Baltimore, MD, USA; 4 Bumrungrad International Hospital, Bangkok, Thailand

**Keywords:** AEC, medical licensing examination, medical qualification, medical education, medical practice

## Abstract

**Background:**

In the regional movement toward ASEAN Economic Community (AEC), medical professions including physicians can be qualified to practice medicine in another country. Ensuring comparable, excellent medical qualification systems is crucial but the availability and analysis of relevant information has been lacking.

**Objective:**

This study had the following aims: 1) to comparatively analyze information on Medical Licensing Examinations (MLE) across ASEAN countries and 2) to assess stakeholders’ view on potential consequences of AEC on the medical profession from a Thai perspective.

**Design:**

To search for relevant information on MLE, we started with each country's national body as the primary data source. In case of lack of available data, secondary data sources including official websites of medical universities, colleagues in international and national medical student organizations, and some other appropriate Internet sources were used. Feasibility and concerns about validity and reliability of these sources were discussed among investigators. Experts in the region invited through HealthSpace.Asia conducted the final data validation. For the second objective, in-depth interviews were conducted with 13 Thai stakeholders, purposely selected based on a maximum variation sampling technique to represent the points of view of the medical licensing authority, the medical profession, ethicists and economists.

**Results:**

MLE systems exist in all ASEAN countries except Brunei, but vary greatly. Although the majority has a national MLE system, Singapore, Indonesia, and Vietnam accept results of MLE conducted at universities. Thailand adopted the USA's 3-step approach that aims to check pre-clinical knowledge, clinical knowledge, and clinical skills. Most countries, however, require only one step. A multiple choice question (MCQ) is the most commonly used method of assessment; a modified essay question (MEQ) is the next most common. Although both tests assess candidate's knowledge, the Objective Structured Clinical Examination (OSCE) is used to verify clinical skills of the examinee. The validity of the medical license and that it reflects a consistent and high standard of medical knowledge is a sensitive issue because of potentially unfair movement of physicians and an embedded sense of domination, at least from a Thai perspective.

**Conclusions:**

MLE systems differ across ASEAN countries in some important aspects that might be of concern from a fairness viewpoint and therefore should be addressed in the movement toward AEC.

The Association of Southeast Asian Nations (ASEAN) is the geo-political and economic cooperation across ten countries: Brunei Darussalam, Cambodia, Indonesia, Laos, Malaysia, Myanmar, the Philippines, Singapore, Thailand, and Vietnam. To promote free trade and services across boundaries, an initiative called the ASEAN Economic Community (AEC) will start in 2015 (1). According to the Mutual Recognition Arrangement (MRA) of this regional movement, physicians, nurses, and dentists are among seven selected professional groups (physician, nurse, dentist, accountant, engineer, architect, and surveyor) that can be qualified to practice in another country (2). The flow of health professionals and cross-border health services is seen as crucial to the success of AEC but needs to be evaluated (3).

Literature on the potential implications of international trade in health services has focused only on the exchange of health care providers and patients across borders or mal-distribution of health resources across urban and rural areas (4, 5). An analysis of current trade patterns based on a ‘four modes of supply’ framework has focused on location and movement of suppliers and consumers; (4) however, this framework may be too simplified for health care professionals, especially physicians. The four modes – cross-border supply, consumption abroad, commercial presence, and movement of individual service providers – did not touch upon the production of suppliers, which is also included in the current context. No framework that integrates both pre- and post-production as well as migration of health care professionals in ASEAN has been proposed in the literature.

Lessons from the European Union (EU) about physician migration could inform the AEC initiative for health care professionals. Health professionals’ migration is affected by push and pull factors (6, 7) as people are more likely to move from their current job with low pay, poor working conditions, as well as limited career opportunities to a relatively better position in another site. Since countries may have different standards, qualifications, and linguistic requirements, there is a period of adapting to clinical, organizational, and social culture of the new country. Unlike non-health care professions, clinical practice relies not only on medical knowledge and skill, but also on interpersonal communication with patients and relatives (8). EU experience suggested that language is one of the most important factors that affect the movement of professional groups, especially in the health care sector (6).

To be a physician, one must complete the required professional medical training, be conferred the professional medical qualification, and be licensed by the Professional Medical Regulatory Authority (PMRA) in the Country of Origin documenting they are technically, ethically, and legally qualified to undertake professional medical practice (2). This privilege usually is not automatically recognized by the ‘Host Country’ (2) – a country where a foreign medical practitioner applies for registration to practice medicine. Australia, for example, requires overseas-trained health professionals to pass fitness-to-practice assessments prior to being registered to practice (9).

Medical education and physician migration are related (10). On the one hand, unequal educational capacity leads to imbalances in the physician workforce (11). On the other hand, a critical mass of physicians is needed to sustain and enhance the medical education enterprise. Further, some countries intentionally train a surplus of health care professionals to supply other countries. Until now, evidence relevant to both AEC and health services were mainly about graduated health professionals but not about medical education and qualification systems.

Current attempts to prepare for the transition to the AEC have focused more on medical education than qualifications (10). The President of the Medical Council of Thailand announced an effort to open more medical schools not only to support the Thai government's medical hub policy but also resolve the doctor shortage problem (12).

Comparable medical qualification systems are crucial to ensure a ‘fair exchange’ of physician workforces among countries. Differences will need to be addressed as part of the effort to harmonize the systems and to realize the MRA and free flow of medical practitioners. Despite the existence of PMRAs in each country (Table 1), good analysis and synthesis of relevant information on medical qualifications in the ASEAN region, has been lacking. The objectives of the present study were (1) to comparatively analyze information on medical licensing examination (MLE) systems across ASEAN countries and (2) to assess stakeholders' view on potential consequences of the AEC on the medical profession from a Thai perspective.

**Table 1:** Professional Medical Regulatory Authority (PMRA) of ASEAN Countries

Member State	Professional Medical Regulatory Authority (PMRA)
Brunei Darussalam	Brunei Medical Board
Cambodia	Cambodian Medical Council and Ministry of Health
Indonesia	Indonesian Medical Council and Ministry of Health
Lao PDR	Ministry of Health
Malaysia	Malaysian Medical Council
Myanmar	Myanmar Medical Council, Ministry of Health
Philippines	Professional Regulation Commission, Board of Medicine and Philippine Medical Association
Singapore	Singapore Medical Council and Specialists Accreditation Board
Thailand	Thailand Medical Council and Ministry of Public Health
Vietnam	Ministry of Health

Source: 2009 ASEAN Mutual Recognition Arrangement on Medical Practitioners. 14th ASEAN Summit, February 26, 2009; Cha-am, Thailand.

## Methods

This study is comprised of two components. To search for relevant information on the MLEs, we included data from the ten national authorities potentially responsible for MLEs of each country as our primary data source. We initially evaluated official websites and made additional queries using email or telephone where possible. Data from these sources were considered most reliable and valid, but were not always available for some countries such as Singapore that does not have centralized MLE, or Brunei that only imports physicians.

For countries lacking a primary data source or with incomplete data, we needed to use data from alternative sources. To gain a better understanding of the national systems, we first checked the official website of medical schools listed in the International Medical Education Directory (IMED) that offered MLE-relevant information (13).

We then approached our colleagues in the Asian Medical Students’ Association (AMSA) – the largest medical student community in Asia (14). This insider's information was quite reliable but might be incomplete. Our third source of data was the Chula-ASEAN Medical Schools Initiative (CU-AMSI) – a collaboration between Chulalongkorn University, Thailand; University of Health Sciences of Cambodia; University of Health Sciences, Laos PDR; University of Medicine 1, Yangon, Myanmar; University of Medicine 2, Yangon, Myanmar; University of Pharmacy, Yangon Myanmar to strengthen the countries’ capacity in medical education and research. Medical students from these countries were informally interviewed to assess their knowledge about MLEs. Some essential information missed by the above approaches was retrieved from other Internet sources as appropriate. Feasibility and concerns about validity and reliability of the data from these secondary sources were discussed among the investigators. The initial synthesis of information was sent to experts in the region, who were invited through HealthSpace.Asia connections. They were asked to validate the findings specific to their countries and then to provide some corrections with supporting evidence.

**Table 2:** Comparing Medical Licensing Examination across 10 ASEAN Countries

		Thailand	Philippines	Singapore	Indonesia	Malaysia	Vietnam	Myanmar	Cambodia	Lao PDR	Brunei
National authority		Center for Medical Competency Assessment and Accreditation	Philippines Board of Medicine	Singapore Medical Council	Indonesia Medical Council	Malaysia Medical Council	Health Ministry/Provincial Agency	Myanmar Medical Council	National Exit Exam Committee and Medical Council of Cambodia	National Medical Council of Laos	Brunei Medical Board
Language in		English 50%	English	English	Bahasa	English		English	Khmer	English,	No
examination		Thai 50%			Indonesia			Burmese		Laos	
Official language		Thai	Philipino English	English, Malaysian, Chinese	Bahasa Indonesia	Malaysian, English	Vietnamese	Burmese	Khmer	Laos	Bahasa Melayu
Steps		3	1	5	1	1		1	1,3	1	0
Methods	MCQ	Yes			Yes	Yes					No
of examination	MEQ	Yes	Yes								No
	OSCE	Yes			Yes	Yes					No
# Medical schools		21	43	2	73	24	12	8	2	1	1
Duration of courses	Pre-clinic	3	3	2	3	2	3	3	6	3	No
(years)	Clinic	3	1	3	3	3	3	3–3.5	2	3	No
	Total	6	5	5	6	5	6	6–7	8	6	No
Doctor:Patient		1:2,700	1:1,800	1:580	1:7,700	1:1,400	1:1,900	1:2,800	1:6,300	1:1,700	1:736
Population (×1,000)		67,312	94,013	5,077	234,181	28,909	86,930	52,797	15,296	6,230	415
Centralization		Yes	No	No	No		No				

For stakeholder analysis, in-depth interviews were conducted with 13 Thai experts. Based on maximum variation sampling technique (15), they were purposively selected to represent the medical licensing authority, the medical profession, ethicists as well as economists. The interview guide contained questions about the impact of AEC on the medical profession, the validity of the medical license, and other important issues relevant to MLE. The interviews were voice recorded and transcribed in Thai language. After all data had been collected, the investigators initially familiarized themselves with the data by listening to tapes and re-reading transcribed interviews in order to identify key ideas and recurrent themes until the investigators became familiar with them in their entirety. A coding scheme was then developed by drawing on a priori issues and questions derived from the study objectives, points raised during the interviews, as well as themes that recurred in the data. The coding scheme was used to code all transcripts. Atlas.ti 6 software (Scientific Software Development GmbH, Berlin, Germany) was used to facilitate the qualitative data analysis.

This study was part of a project submitted to a Medical Ethics course, Faculty of Medicine, Chulalongkorn University. The two components of this study had minimal ethical concerns and were not submitted for consideration by the Institutional Review Board (IRB). The first part of this study deals with only publicly available information. Although the second part of this project involved subjects who were key informants, data was ‘from’ them but not ‘about’ them. Rather, it was opinions and judgments about MLE system from a Thai perspective. Therefore, the project did not qualify as human subjects research as defined by United States Department of Health and Human Services regulations 45 CFR 46.102, and did not require IRB review, which also concurs with relevant Thai regulations.

## Results

Brunei has the best doctor to patient ratio despite no medical school or MLE (Table 2). In most countries, MLEs are run by their respective medical council, which usually is a part of the country's government health ministry. With regard to length of time of recognition of qualification, some countries, including Thailand, offer life-long certification, whereas Vietnam requires a renewal every 5 years.

Thailand and Indonesia have a national MLE system whereas Singapore and Vietnam accept results of MLE conducted at universities. Although the MLE of most countries requires only one step, Thailand adopts the USA's 3-step approach that aims to check pre-clinical knowledge, clinical knowledge, and clinical skills. In Cambodia, the MLE system is comprised of two components. The National Exit Exam Committee is responsible for organizing the national examination and the Medical Council of Cambodia will provide a license to practice to medical doctors who pass the examination.

Regarding examination types, multiple choice question (MCQ) formats are the most commonly used, followed by the modified essay question (MEQ) format. Although both tests try to assess a candidate's knowledge, the Objective Structured Clinical Examination (OSCE) was used to verify clinical skills of the examinee.

Medical education systems vary across ASEAN countries and affect the development of MLE systems. Medical students usually were required to complete 5–7 years of coursework. In Singapore, medical students must pass an annual examination in their medical schools before they can proceed to the next level. Immediately following successful completion of the fifth-year examination, they receive provisional registration which is valid only for the internship period; successful completion of the internship allows progression to full registration. This 5-step MLE system is similar to Thailand's system before centralization.

Language variation across countries does exist not only for general communication, but also in the MLE. English is used in seven countries whereas six prefer the local language, especially in the OSCE. Although Thailand's official language is Thai, approximately 10% of the written examinations for pre-clinical and clinical knowledge are in English. Similar phenomena occur in Myanmar, Cambodia, and Laos where English examination questions are added to the ones in their respective native languages. Interestingly, Malaysia uses only English rather than its native language. Vietnam and Indonesia are the only countries that do not use the English language in its MLE at any stage.

Three major themes emerged from in-depth interviews with 13 Thai experts. The first theme is the extent to which a medical license issued by one country is valid in other countries. Two interesting concepts emerged from the interviews. ‘License transfer’ happens when a license issued by country A is valid in both country A and B. This, from the interviewee's perspective, seems to result in a sense of domination by country A. ‘Common license’ means a license jointly issued by and therefore valid in all participating countries. Although this common license forms a sense of community, only 2 out of 13 interviewees thought that it is possible to achieve; the rest disagreed mainly because of diverse systems and contexts across ASEAN countries in the quality of medical education, language used, and culture. ‘Each country has its own rule and context, to which whoever would like to do clinical practice must comply. As the country systems have been developed to promote the national interest, not regional, I consider an ASEAN common license unethical’, stated one interviewee. All interviewees still thought that further development of medical education and practice in this region is essential.

The second theme is about the language used in each step of the licensing examination. Ten out of thirteen interviewees said basic science and clinical knowledge examination should be executed in a common language like English. Experts from all but the economic field stressed the importance of using native language in assessing clinical competency not only must knowledge and skill be evaluated but also interpersonal communication. ‘It would be unfair to a Thai patient if his or her doctor does not speak the same language. Drug prescription and surgical operation are important, but the patient would be worse off without a clear understanding of what the doctor tried to explain’.

The third theme is that migration of both supply and demand of health care services is complex. Although many interviewees voiced a concern about the migration of Thai physicians to places with better financial benefits, seven thought that non-financial aspects of the Thai context may be more influential including returning to Thailand for family or social reasons. On the contrary, the movement of both medical practitioners and patients from other countries into Thailand is more likely but the impact on health systems is inconclusive.

## Discussion

This study is the first to offer comparative information on medical qualification systems across ASEAN countries. In addition to different basic characteristics of medical education, MLE systems in ASEAN countries are diverse in many aspects including language, number of steps in the process, as well as methods of examination. These differences need to be addressed as part of the harmonization effort. Findings from an online survey suggested that ‘recognition of qualification’ should be standardized and could start from basic functions such as licensure/registration, especially when evidence on the competence of regulators and their diversity were still unclear (16).

The initial assumption that information on medical education and MLE systems would be readily accessible from a country's national body turned out to be invalid as our attempts to identify a primary source revealed incomplete information. Secondary sources became important for our data analysis and synthesis despite questionable quality when standard criteria were applied (17). The objective of this study was just to compare the systems in general.

Unlike individuals in other professions, health care professionals, especially doctors, require not only technical but also interpersonal skills. Evidence suggested that quality of care can be adversely affected by a language barrier (18, 19). In addition to language, evidence consistently suggested that race and ethnicity also substantially influenced the quality of the doctor-patient relationship (20). Validity of the medical license is a sensitive issue, at least from the Thai perspective. The potential imbalance or unfair movement of physicians and relevant policies or agreements across ASEAN countries embed a sense of domination (21); this inevitably distorts the concept of unity. Any political negotiation regarding the medical license validity should be carefully done, however.

Currently, there are some attempts to unify the medical curriculum and the examination system across India (22) and the Emirates (23) to diminish regional differences; however, the application of these experiences to ASEAN region without adequate data is limited. Information presented in this study is beneficial for potential harmonization of medical education and qualification systems across national boundaries. We hope that the comparative data and the Thai perspective presented in this study will provide input for the collaborative development of a framework for smooth implementation of relevant systems to facilitate free movement of doctors across ASEAN countries under the Mutual Recognition Arrangements. As only Thai perspective was focused in the present study, further harmonized efforts should be done by representatives of all ASEAN countries to synthesize country specific concerns.

## Conclusion

MLE systems differ across ASEAN countries in some important aspects that might be of concern from a fairness viewpoint and therefore should be addressed in the movement toward AEC.
